# Supplementary material for: Content-rich biological network constructed by mining PubMed abstracts
Source: BMC Bioinformatics. 2004 Oct 8;5:147. doi: 10.1186/1471-2105-5-147 (PMC528731; doi:10.1186/1471-2105-5-147)
Supplement: Additional File 2 — The original results of the above study (non-essential files are deleted to keep the file size under the limit set by BMC bioinformatics). [file 1471-2105-5-147-S2.bz2 › chilibotAdditionalFile2/dip05/655ID9083111E11046/html/GCTA_GCTB.html]

 


 **GCTA** and **GCTB** 
  
Found 2 abstracts in PubMed, retrieved 2.  
 

 What does Google say? 
 PDF only 
| .edu only 

---

**Interactive relationship** (e.g. stimulation, inhibition, etc)

**Non-interactive relationship** (e.g. studied together, co-existance, homology, etc.)

- A 65 kDa protein, obtained by fusing the genes,  **gctA**   **gctB** , coding for glutaconate CoA transferase, retained 30% of the wild type activity.  Ref: 9089292 FEBS Lett, 1997
- Glutaconate coenzyme A transferase Gct from Acidaminococcus fermentans consists of two subunits  **GctA** , 35725 Da and  **GctB** , 29168 Da.  Ref: 7957258 Eur J Biochem, 1994
